# Three-dimensional-printed strontium-incorporated β-TCP bioceramic triply periodic minimal surface scaffolds with enhanced angiogenic and osteogenic properties

**DOI:** 10.1093/rb/rbaf080

**Published:** 2025-08-12

**Authors:** Yanbo Shan, Yang Bai, Lisheng Zhao, Qing Zhou, Shuo Yang, Gang Wang, Ye Lei, Yuzheng Lu, Yanbin Wu, Yu Wei, Jiang Peng, Rujie He, Ning Wen, Bin Gu

**Affiliations:** Graduate School of the PLA General Hospital, Beijing 100853, China; Institute of Stomatology & Oral Maxilla Facial Key Laboratory, First Medical Center of Chinese PLA General Hospital, Beijing 100853, China; Institute of Orthopedics, Chinese PLA General Hospital, Beijing Key Lab of Regenerative Medicine in Orthopedics, Key Laboratory of Musculoskeletal Trauma & War Injuries PLA, Beijing 100853, China; Institute of Stomatology & Oral Maxilla Facial Key Laboratory, First Medical Center of Chinese PLA General Hospital, Beijing 100853, China; Institute of Stomatology & Oral Maxilla Facial Key Laboratory, First Medical Center of Chinese PLA General Hospital, Beijing 100853, China; Institute of Advanced Structure Technology, Beijing Institute of Technology, Beijing 100081, China; Institute of Stomatology & Oral Maxilla Facial Key Laboratory, First Medical Center of Chinese PLA General Hospital, Beijing 100853, China; Institute of Stomatology & Oral Maxilla Facial Key Laboratory, First Medical Center of Chinese PLA General Hospital, Beijing 100853, China; Institute of Stomatology & Oral Maxilla Facial Key Laboratory, First Medical Center of Chinese PLA General Hospital, Beijing 100853, China; Institute of Orthopedics, Chinese PLA General Hospital, Beijing Key Lab of Regenerative Medicine in Orthopedics, Key Laboratory of Musculoskeletal Trauma & War Injuries PLA, Beijing 100853, China; Institute of Orthopedics, Chinese PLA General Hospital, Beijing Key Lab of Regenerative Medicine in Orthopedics, Key Laboratory of Musculoskeletal Trauma & War Injuries PLA, Beijing 100853, China; Institute of Orthopedics, Chinese PLA General Hospital, Beijing Key Lab of Regenerative Medicine in Orthopedics, Key Laboratory of Musculoskeletal Trauma & War Injuries PLA, Beijing 100853, China; Institute of Orthopedics, Chinese PLA General Hospital, Beijing Key Lab of Regenerative Medicine in Orthopedics, Key Laboratory of Musculoskeletal Trauma & War Injuries PLA, Beijing 100853, China; Institute of Advanced Structure Technology, Beijing Institute of Technology, Beijing 100081, China; Institute of Stomatology & Oral Maxilla Facial Key Laboratory, First Medical Center of Chinese PLA General Hospital, Beijing 100853, China; Institute of Stomatology & Oral Maxilla Facial Key Laboratory, First Medical Center of Chinese PLA General Hospital, Beijing 100853, China

**Keywords:** strontium, β-tricalcium phosphate, triply periodic minimal surface, osteogenesis, angiogenesis

## Abstract

Reconstructing bone defects remains a significant challenge in clinical practice, driving the urgent need for advanced artificial grafts that simultaneously promote vascularization and osteogenesis. Addressing the critical trade-off between achieving high porosity/strength and effective bioactivity at safe ion doses, we incorporated strontium (Sr) into β-tricalcium phosphate (β-TCP) scaffolds with a triply periodic minimal surface (TPMS) structure using digital light processing (DLP)-based three-dimensional (3D) printing. Systematically screening Sr concentrations (0–10 mol%), we identified 10 mol% as optimal, leveraging the synergy between the biomimetic TPMS architecture, providing exceptional mechanical strength (up to 1.44 MPa at 80% porosity) and facilitating cell recruitment and precision Sr-dosing to enhance bioactivity. *In vitro* assays revealed that the Sr-TCP scaffold dose-dependently stimulated osteogenic differentiation and mineralization in mouse osteoblastic cell line (MC3T3-E1) cells, while also significantly enhancing the angiogenic capacity in human umbilical vein endothelial cells (HUVECs). *In vivo* studies indicated that the scaffold demonstrated synergistic osteogenic and angiogenic effects in rat femoral condylar defects, leading to marked improvements in bone healing. Collectively, this study establishes a novel design paradigm combining biomimetic topology with optimized ionic doping, resolving key limitations of conventional grafts and advancing the development of safe, highly effective biomaterials for vascularized bone regeneration.

## Graphical abstract

**Figure:**
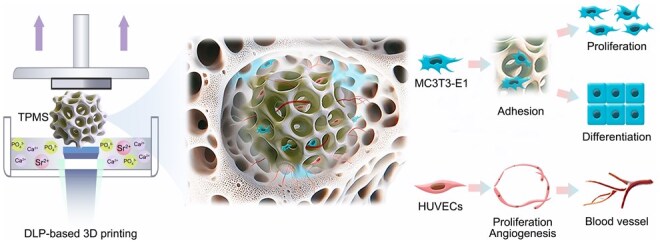

## Introduction 

Bone defect treatment remains a significant clinical challenge, with pathological conditions like trauma, infection and tumors further complicating the healing process [1]. While bone grafting—autologous, allograft and xenograft—is a widely used restoration strategy, its application is limited by risks such as immune rejection and postoperative infection [2]. In recent years, bone tissue engineering (BTE), which integrates biological scaffolds, stem cells and growth factors, has emerged as a groundbreaking approach to address bone deficiencies, offering a promising alternative to traditional grafting methods [3]. A key principle in orthopedic surgeries is vascularized osteogenesis, as bone is a highly vascularized tissue where the interplay between angiogenesis and osteogenesis is crucial for development, remodeling and regeneration [4]. Consequently, there is a pressing need to develop advanced biomedical scaffolds within BTE that exhibit superior osteogenic and angiogenic properties to promote vascularized bone regeneration. However, achieving scaffolds that simultaneously offer high porosity for cell/vessel ingrowth, sufficient mechanical strength for defect support and potent bioactivity at safe ion concentrations represents a fundamental challenge.

Calcium phosphate (Ca_3_(PO_4_)_2_, TCP) is a globally recognized bioactive scaffold [5], yet its clinical application, particularly in large defects, faces hurdles due to insufficient vascularization and limited osteogenesis. Current strategies to enhance TCP primarily focus on two key approaches: structural optimization and the integration of biofunctionalized dopants. Innovative structural modifications, such as honeycomb-type macroporous designs [6], hierarchically organized porous frameworks [7] and nanoscale whisker configurations [8], have been developed, significantly improving new vessel and bone formation. Concurrently, the incorporation of bioactive ions has shown remarkable efficacy. Wei *et al*. engineered a nano-zinc oxide particle-enhanced hydroxyapatite whisker, providing significant advancements in creating bone substitutes with enhanced osteoinductive capabilities [9]. Extensive research underscores the pivotal role of scaffold structural parameters in cell adhesion and bone ingrowth, while bioactive ions released from these scaffolds activate signaling pathways crucial for cell proliferation and differentiation [10, 11]. Despite progress, simultaneous optimization across structural and compositional dimensions remains underexplored, especially for achieving vascularized regeneration with minimal bioactive ion doses to mitigate toxicity risks.

High porosity is essential for facilitating cell ingrowth and ensuring the continuous supply of oxygen and nutrients through vascular networks. In recent years, the triply periodic minimal surface (TPMS) structure, which replicates the hyperboloidal geometry of trabecular bone, has garnered significant attention in the development of biological porous scaffolds. Known for its stable and reliable mechanical properties—such as elastic modulus, compressive stress and anisotropic behavior—the TPMS structure derives its strength from constant-mean-curvature surfaces [12]. Biologically, its high porosity and interconnected pore network enhance stem cell permeability, promoting vascularization in surrounding tissues [13]. Additionally, the hyperboloid topology has been shown to induce nuclear deformation and cytoskeleton reorganization in stem cells, thereby accelerating bone regeneration [14]. Recent studies have increasingly demonstrated the superior structural and biological advantages of TPMS-based scaffolds in bone regeneration applications [15, 16]. However, the intricate topology of TPMS structures poses challenges for precise replication using conventional methods. Advances in three-dimensional (3D) printing, particularly digital light processing (DLP), have revolutionized the fabrication of TPMS structures across diverse scales and materials, offering enhanced efficiency and precision [17, 18]. This technological leap has opened new avenues for the production of TPMS-based porous scaffolds.

The integration of bioactive ions into biological scaffolds has been shown to significantly enhance the healing process [19–21]. Among these, strontium (Sr), a trace element predominantly found in bone tissue, is particularly notable for its dual role in promoting osteoblast differentiation and inhibiting osteoclast resorption [22]. Recent studies further suggest that sustained Sr release can stimulate local angiogenesis [23]. However, excessive Sr^2+^ accumulation could disrupt mineral and collagen fibril assembly, potentially leading to bone-weakening disorders, as highlighted by Bussola Tovani *et al*. [24]. Although numerous studies have reported that strontium-doped biomaterials accelerate bone repair [25, 26], the typically high Sr content raises concerns over potential risks. Thus, a core scientific question emerges: Can the synergistic combination of a biomimetic TPMS structure and precision Sr-doping enable effective vascularized bone regeneration at significantly reduced and safer Sr doses? We hypothesize that integrating low-dose Sr incorporation with the mechanically robust and biologically favorable TPMS-structured TCP scaffolds will synergistically enhance vascularized osteogenesis while minimizing Sr-related risks, a strategy not yet explored to address the dose-efficacy-safety trade-off.

**Figure 1.**
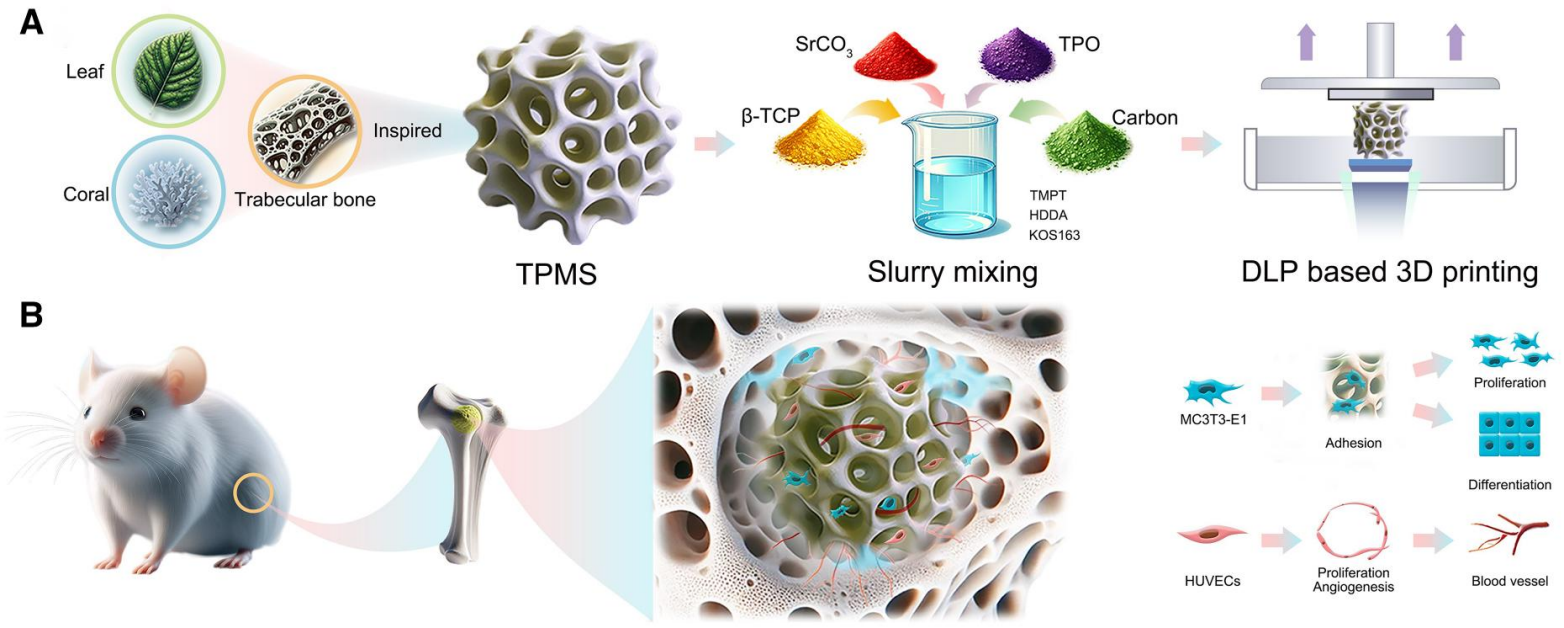
Schematic representation of the fabrication and biological function of Sr-incorporated β-TCP bioceramic TPMS scaffolds. (**A**) Inspired by the microstructure of leaves, coral and trabecular bone, the scaffolds were fabricated using a DLP-based 3D printing technique. (**B**) These scaffolds modulate cellular behaviors, including adhesion, osteogenesis and angiogenesis, to promote vascularized bone regeneration. MC3T3-E1, mouse osteoblastic cell line; HUVECs, human umbilical vein endothelial cells.

To our knowledge, our previous study [27] marked the first successful fabrication of 5 mol% Sr-incorporated β-tricalcium phosphate (β-TCP) bioceramic TPMS scaffolds, which exhibited exceptional mechanical strength and bioactivity for bone regeneration. Building upon this foundation, we employed DLP-based 3D printing to systematically investigate Sr concentration gradients (0, 2.5, 5.0, 10.0 mol%) in TPMS-structured β-TCP scaffolds for the first time, aiming to identify the optimal Sr dose for vascularized bone regeneration (Figure 1). Initial characterization focused on macrostructure, microstructure, phase composition, mechanical properties and *in vitro* degradation behavior of the scaffolds. Subsequently, we systematically evaluated cell viability, adhesion and migration, while comparing the angiogenic and osteogenic properties of scaffolds with different Sr contents *in vitro*, thereby identifying the optimal Sr incorporation concentration. To further validate these findings, we established a rat femoral condylar defect model, which comprehensively demonstrated the enhanced vascularized bone regeneration potential of Sr-incorporated β-TCP bioceramic TPMS scaffolds *in vivo*.

## Materials and methods

### Materials

The ceramic powders utilized in this study included commercial β-TCP from Sinopharm Chemical Reagent Co., Ltd, China, and SrCO_3_ sourced from Shanghai Aladdin Biological Technology Co., Ltd, China. Photosensitive resin monomers comprised 1,6-hexanediol diacrylate (HDDA) and trimethylolpropane triacrylate (TMPTA), both supplied by Sinopharm Chemical Reagent Co., Ltd, China. To ensure optimal ceramic slurry performance, KOS163 from Lubrizol Management (Shanghai) Co., Ltd, China, was employed as the dispersant. The photoinitiator selected was diphenyl (2,4,6-trimethyl-benzoyl) phosphine oxide (TPO) from RYOJI, Germany. Additionally, carbon black from Shanghai Macklin Biochemical Technology Co., Ltd, China, was incorporated to enhance printing accuracy.

### Fabrication of Sr-doped TCP scaffolds

The Sr-doped TCP scaffolds were fabricated using DLP-based 3D printing, as previously described [28]. The 3D TPMS structural models were initially designed using Rhino software, with a controlled porosity of 80% and pore sizes ranging from 300 to 700 μm. Then, varying amounts of SrCO_3_ (0, 2.5, 5.0, 10.0 mol%) were incorporated into β-TCP powders, followed by chemical reactions. A mixture of HDDA and TMPTA was used to dissolve the ceramic powders at a solid loading of 30 vol%. Subsequently, TPO photoinitiator, KOS163 dispersant and carbon black were added and ball-milled in a planetary mill (QM-3SP2, Nanjing University Instrument Plant, China). The composition of each component in the Sr-doped TCP scaffolds is detailed in Table 1. The scaffolds were then fabricated using a DLP 3D printer (AutoCera, Beijing 10dim Tech. Co., Ltd, China) based on 3D Gyroid TPMS structure models, with the following parameters: initial layer exposure time = 8 s, subsequent layer exposure time = 7 s, layer thickness = 50 μm and light intensity = 19 000 μW/cm^2^. For *in vitro* experiments, the scaffolds were cylindrical with a diameter of 5 mm and a height of 5 mm; for *in vivo* experiments, the dimensions were 2.5 mm in diameter and 3 mm in height. The green bodies were sintered in a muffle furnace (Hefei Facerom Furnace Co., Ltd, China) at 1250°C for 100 min to produce the final bioceramic scaffolds. The samples were designated as TCP, 2.5Sr-TCP, 5Sr-TCP and 10Sr-TCP.

**Table 1.** Components for Sr-doped β-TCP.

Components	TCP	2.5Sr-TCP	5Sr-TCP	10Sr-TCP
Ca_3_(PO_4_)_2_ (g)	142.2	137.9	133.63	125.27
SrCO_3_ (g)	0	5.1	10.03	19.82
HDDA (g)	84.84	84.84	84.84	84.84
TMPTA (g)	23.27	23.27	23.27	23.27
TPO (g)	2.16	2.16	2.16	2.16
KOS163 (g)	5.51	5.51	5.51	5.51
C (g)	0.71	0.72	0.72	0.73

### Characterization of Sr-doped TCP scaffolds

The physical structure of the TCP and Sr-doped TCP scaffolds was examined using a scanning electron microscope (SEM, JSM-7500F, Japan), while their elemental distribution and composition were analyzed via an energy diffraction spectrometer (EDS, Quantax EDS, Bruker, Germany). The phase composition of the scaffolds was analyzed using X-ray powder diffraction (XRD, Bruker D8 Advance, Bruker Co., Germany). XRD patterns were collected in the 2θ range of 10–80° with a step size of 0.033°. Compressive stress-strain analysis was conducted using a universal mechanical testing machine (Instron Legend 2367 testing system, USA) at a crosshead speed of 0.05 mm/min. Each experiment was performed in triplicate to ensure reliability. To assess *in vitro* scaffold degradation and ion release profiles, each scaffold sample (70 mg) was immersed in 7 mL of simulated body fluid (SBF, Phygene, China) and incubated in a 37°C cell culture incubator. The dry weight of the remaining scaffolds was measured at weeks 1, 2 and 3. The soaking solution was collected on Days 1, 3, 5 and 7 for Ca^2+^ and Sr^2+^ concentration measurements using inductively coupled plasma-mass spectrometry (ICP-MS, Agilent 7800, USA), and 7 mL of fresh SBF was replenished to continue the release test.

### Cell cultivation and extract preparation

The mouse osteoblastic cell line (MC3T3-E1) cells and human umbilical vein endothelial cells (HUVECs), sourced from the National Collection of Authenticated Cell Cultures (China), were utilized for *in vitro* studies. MC3T3-E1 cells were maintained in alpha-modified eagle medium (α-MEM, Gibco, USA) enriched with 10% fetal bovine serum (Gibco, USA) and 1% penicillin-streptomycin-amphotericin B solution (Gibco, USA). Similarly, HUVECs were cultured in endothelial cell medium (ECM) under identical conditions at 37°C in a 5% CO_2_ humidified incubator.

Sample extracts were prepared following ISO 10993-12:2021 guidelines by incubating Sr-doped TCP scaffolds in α-MEM or ECM at a ratio of 0.1 g/mL for 72 h at 37°C in a humidified environment. These extracts were subsequently diluted to a 10% concentration with the respective medium for subsequent experimental procedures.

### Cell proliferation, viability and spreading assessment

MC3T3-E1 cells and HUVECs were cocultured with extracts from different groups, with MEM/ECM mediums serving as the negative control. Cell proliferation was assessed using the Cell Counting Kit-8 (CCK-8, Dojindo, Japan) at 1, 3 and 5 days, and optical density (OD) values were measured at 450 nm using a microplate spectrophotometer (BioTek, UK). After 3 days of coculture, cell viability was evaluated using the Calcein AM and PI staining kit (Beyotime, China), with live (green) and dead (red) cells visualized under a stereomicroscope (SMZ25, Nikon, Japan). For cytoskeleton immunofluorescent staining, MC3T3-E1 cells and HUVECs were fixed with 4% paraformaldehyde (Biorigin, China), followed by staining with rhodamine-phalloidin (Bioscience, China) for 30 min and Dapi (Servicebio, China) for 5 min at room temperature. Images were captured using a pannoramic confocal microscope (Pannoramic, 3DHISTECH, Hungary).

MC3T3-E1 cells were seeded onto Sr-doped TCP scaffolds and cultured for 48 h. Following overnight fixation with 2.5% glutaraldehyde (Sigma-Aldrich, USA) at 4°C, the scaffolds underwent dehydration using a graded ethanol series (Sigma-Aldrich, USA). After drying, the scaffolds were gold-coated, and cell morphology was visualized using a biological SEM (Hitachi H-7650, Japan).

### Migration ability and osteogenesis assessments of MC3T3-E1 cells

When MC3T3-E1 cells achieved 100% confluency, 200 μL pipette tips were used to create uniform scratches through the center of each well. Following a 12-h coculture with 2%-serum extracts across various groups, the scratch areas were imaged using a stereomicroscope (SMZ25, Nikon, Japan). The wound healing rates were subsequently quantified using Image Pro Plus 6.0 software.

The osteogenic induction medium (OM) was prepared by supplementing α-MEM with 10 mM β-glycerol phosphate, 100 nM dexamethasone and 50 mg/mL ascorbate-2-phosphate, and was refreshed every 3 days throughout the osteoinduction process. Alkaline phosphatase (ALP) staining was conducted using a BCIP/NBT staining kit (Beyotime, China) following 7 and 14 days of coculture with osteogenic extracts. Stained cells were visualized using a stereomicroscope (SMZ25, Nikon, Japan). ALP activity was quantified using the Alkaline Phosphatase Assay Kit (Beyotime, China), with protein content determined by the enhanced BCA Protein Assay Kit (Beyotime, China) for normalization. Calcium nodules formed after 21 days of osteogenic induction were stained with Alizarin Red S Solution (Solarbio, China) and imaged using the stereomicroscope (SMZ25, Nikon, Japan). The stained calcium deposits were dissolved with 10% cetylpyridinium chloride (CPC, Sigma-Aldrich, USA), and mineralization was quantified by measuring OD values at 562 nm using a microplate spectrophotometer (BioTek, UK).

### Migration ability and tube formation assessments of HUVECs

HUVECs were plated in the upper chamber of an 8 μm-pore transwell system (Corning, USA), with sample extracts from different groups introduced into the lower chamber. After 24 h, cells that migrated to the lower surface of the insert were fixed using 4% paraformaldehyde (Biorigin, China) for 30 min and subsequently stained with 0.1% crystal violet (Solarbio, China). Images were acquired using a laser confocal microscope (LSM780, Zeiss, Japan).

For the tube formation assay, 10 μL of Matrigel (Corning, USA) was uniformly coated onto a µ-Slide 15 Well 3D (Ibidi, Germany) and incubated at 37°C for 30 min to facilitate gelation. HUVECs were then seeded onto the Matrigel and exposed to various extracts for 4 h in a cell incubator. Post-incubation, tube formation was visualized using an optical microscope (Olympus, Japan), and quantitative analysis of tube length, node count and junctions was performed using Image Pro Plus 6.0 software.

### Immunofluorescence staining

MC3T3-E1 cells were cocultured with α-MEM extracts across different groups for 48 h, then, fixed in 4% paraformaldehyde (Biorigin, China) for 10 min. After blocking with goat serum working solution (1:20, Solarbio, China) for 30 min, the samples were incubated overnight at 4°C with a primary antibody against vinculin (1:100, Abcam, UK). The following day, cells were treated with a fluorescent secondary antibody (1:150, Abcam, UK) for 2 h, and nuclei were stained with Dapi (Servicebio, China) for 5 min. Imaging was conducted using a confocal panoramic scanner (Pannoramic, 3DHISTECH, Hungary), with fluorescence intensity quantified via Image Pro Plus 6.0 software. Additionally, immunofluorescence staining for RUNX2 (1:100, Proteintech, China) and OCN (1:100, Proteintech, China) in MC3T3-E1 cells was performed using the same protocol following osteoinduction for 7 or 14 days.

### Quantitative PCR

The osteogenic target genes, including collagen 1 (*Col1*), alkaline phosphatase (*Alp*), osteocalcin (*Ocn*), osteopontin (*Opn*) and runt-related transcription factor 2 (*Runx2*), were analyzed using primer sequences synthesized by Sangon Biotech (Shanghai). Primer details are provided in Table 2. Following osteogenic extract stimulation for 3 and 7 days across different groups, qPCR assays were performed according to the manufacturer’s protocol. Total RNA was isolated from MC3T3-E1 cells using the FastPure Cell/Tissue Total RNA Isolation Kit (Vazyme, China) and reverse transcribed into cDNA with 5×RT Master Mix (TOYOBO, Japan). Gene expression quantification was conducted using 2×RealStar Fast SYBR qPCR Mix (Genstar, China) on a StepOne Real-Time PCR System (Applied Biosystems, China). Gene expression levels were normalized to *Gapdh* and calculated using the 2^−ΔΔCt^ method.

**Table 2.** Primer sequences used for qPCR.

Gene	Forward primer sequence (5'-3')	Reverse primer sequence (5'-3')
*Col1*	GGGACCAGGAGGACCAGGAAGT	GGAGGGCGAGTGCTGTGCTTT
*Opn*	AGCAAGAAACTCTTCCAAGCAA	GTGAGATTCGTCAGATTCATCCG
*Alp*	ATCTTTGGTCTGGCTCCCATG	TTTCCCGTTCACCGTCCAC
*Ocn*	CCAAGCAGGAGGGCAATA	TCGTCACAAGCAGGGTCA
*Runx2*	GAACCAAGAAGGCACAGACAA	GGCGGGACACCTACTCTCATAC
*Gapdh (mus musculus)*	GTATTGGGCGCCTGGTCACC	CGCTCCTGGAAGATGGTGATGG
*VEGF*	CCACGACAGAAGGAGAGCAGAAG	GGTCTCAATCGGACGGCAGTAG
*HIF1*	TGCCACTGCCACCACAACTG	TGCCACTGTATGCTGATGCCTTAG
*HGF*	CAGCATGTCCTCCTGCATC	TCTTTTCCTTTGTCCCTCTGC
*PECAM*	AAGTGGAGTCCAGCCGCATATC	ATGGAGCAGGACAGGTTCAGTC
*VWF*	CCTTGAATCCCAGTGACCCTGA	GGTTCCGAGATGTCCTCCACAT
*GAPDH (homo sapiens)*	GTCTCCTCTGACTTCAACAGCG	ACCACCCTGTTGCTGTAGCCAA

The expression levels of key angiogenesis-related genes, including vascular endothelial growth factor (*VEGF*), hypoxia-inducible factor 1 (*HIF1*), hepatocyte growth factor (*HGF*), platelet endothelial cell adhesion molecule (*PECAM1/CD31*) and von Willebrand factor (*VWF*), were quantitatively assessed using qPCR. Following a 3-day coculture period of HUVECs with various sample extracts, qPCR analysis was performed according to the established protocol. The corresponding primer sequences are detailed in Table 2.

### Animal surgery

Thirty male Sprague Dawley (SD) rats, aged 8 weeks and weighing 250–300 g, were utilized in this study. All experimental protocols received approval from the Experimental Animal Ethics Committee of PLA General Hospital (approval number: 2022-X18-133), ensuring adherence to ethical and welfare standards. The rats were randomly allocated into five groups: (1) defect control, (2) TCP scaffold, (3) 2.5Sr-TCP scaffold, (4) 5Sr-TCP scaffold and (5) 10Sr-TCP scaffold. Anesthesia was induced via intraperitoneal injection of 3% pentobarbital sodium (30 mg/kg), followed by surgical site sterilization with 70% ethanol. A midline sagittal incision was made on the right femoral skin to expose the femoral condyle, and a cylindrical bone defect (2.5 mm in diameter, 3 mm in depth) was created. The scaffold was then implanted into the defect, and the incision was sutured. Post-transplantation, rats were euthanized at 4 and 8 weeks, and femurs were harvested and fixed in 4% paraformaldehyde for subsequent analysis.

### Radiological analysis

All femoral samples were scanned using a Micro-Computed Tomography (Micro-CT) system (SkyScan 1276, BRUKER, Germany) at 80 kV and 200 μA. The acquired images, with a pixel resolution of 8 μm, were reconstructed using NRecon 1.7 software and analyzed with CTAn 1.17 software. The bone defect area was designated as the region of interest (ROI), and key parameters such as the new bone volume relative to total volume (BV/TV) and bone mineral density (BMD) were quantified at the femoral condylar defect site.

### Histological evaluation

The collected samples were decalcified in 10% ethylene diamine tetraacetic acid (EDTA) solution (Solarbio, China) for six weeks. Following decalcification, the tissues were embedded in paraffin, and serial sections of 5 μm thickness were prepared for staining. The sections were stained with hematoxylin & eosin (H&E) and Masson’s trichrome (MT) using the manufacturer’s protocol (Servicebio, China). For immunohistochemistry, the slides were dewaxed, rehydrated and incubated overnight at 4°C with primary antibodies against OPN (1:100, Servicebio, China) and CD31 (1:100, Servicebio, China). The next day, the slides were treated with secondary antibodies (1:150, Servicebio, China) for 1 hr, followed by diaminobenzidine (DAB) (Servicebio, China) for visualization. Nuclei were counterstained with hematoxylin (Servicebio, China) for 3 min. Histological images of the stained sections were captured using a Pannoramic confocal microscope (3DHISTECH, Hungary).

### Statistical analysis

All experimental data were presented as mean ± standard deviation from a minimum of three independent replicates. Statistical analyses were performed using GraphPad Prism 9.4.1 software. Pairwise comparisons were conducted using Student’s *t*-test, while group comparisons were analyzed through one-way analysis of variance (ANOVA). Statistical significance was denoted as follows: NS (non-significant) or significant differences (*P < 0.05, **P < 0.01, ***P < 0.001, ****P < 0.0001 versus control group; ^#^*P* < 0.05, ^##^*P* < 0.01, ^###^*P* < 0.001, ^####^*P* < 0.0001 among experimental groups).

## Results

### Characterization of Sr-doped TCP scaffolds

**Figure 2.**
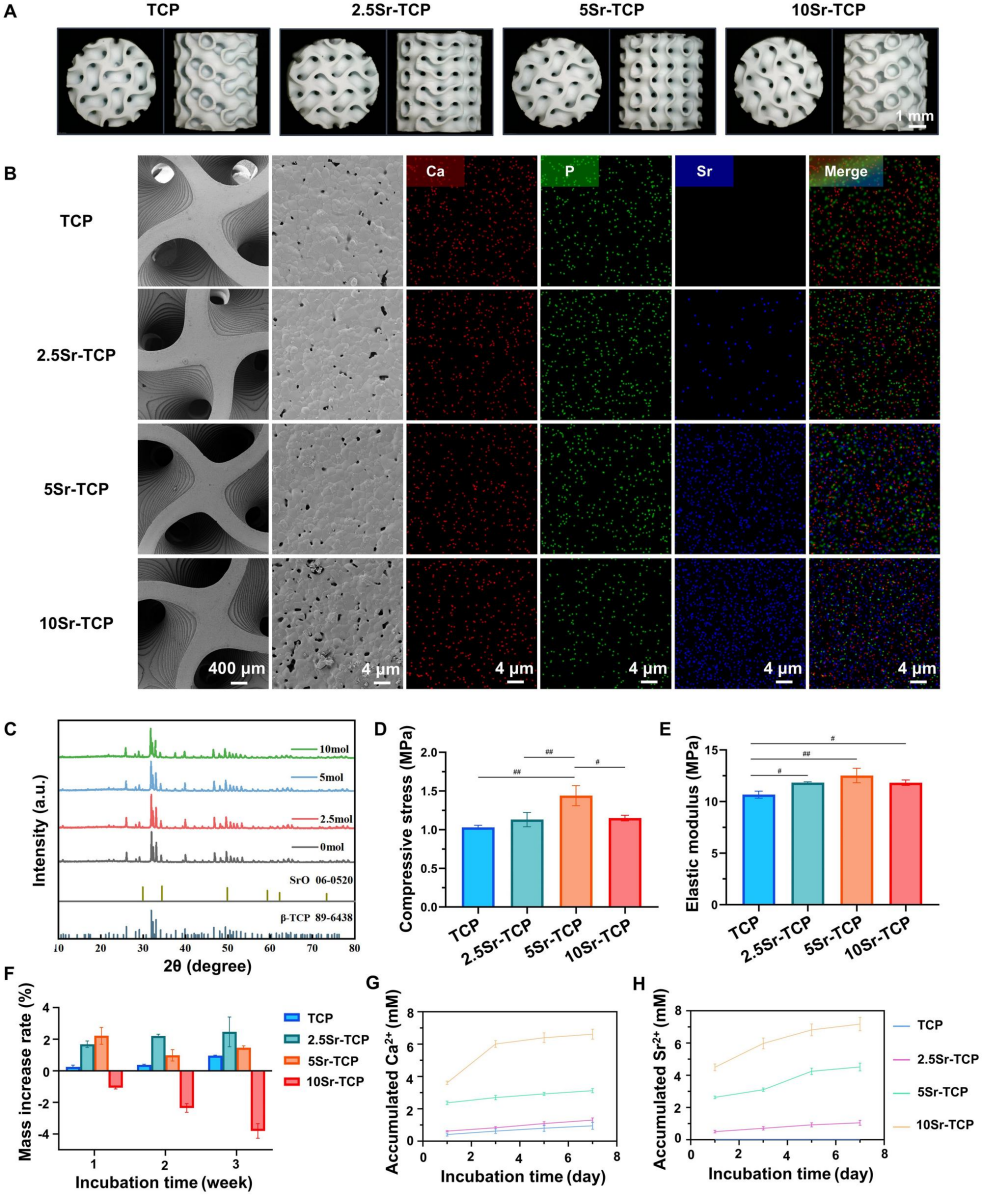
Characterization of TCP and Sr-TCP scaffolds. (**A**) Macroscopic images. (**B**) SEM-EDS mapping results. (**C**) XRD curves and the corresponding standard spectra of each component. (**D**) Compressive stress and (**E**) elastic modulus of the scaffolds. (**F**) Mass increase rate, (**G**) Ca^2+^ accumulation concentration and (**H**) Sr^2+^ accumulation concentration curves of the scaffolds immersed in SBF. (*n* = 3; ^#^*P* < 0.05, ^##^*P* < 0.01 among experimental groups).

The macroscopic morphology of TPMS-structured scaffolds is illustrated in Figure 2A. Figure 2B presents SEM-EDS mapping of TCP and Sr-doped TCP scaffolds, revealing the microstructure and spatial distribution of Sr, Ca and P. In contrast to the undoped TCP, which exhibited only Ca and P, the Sr-doped scaffolds demonstrated uniform Sr dispersion, confirming its successful integration. Quantitative EDS data detailing the elemental composition of the scaffolds are summarized in Table 3. The structural composition of β-TCP scaffolds with varying Sr doping concentrations was analyzed using XRD (Figure 2C). The diffraction patterns confirmed the presence of β-TCP crystalline phases, indicating that strontium incorporation did not change the crystallographic structure. Although the XRD patterns showed weak SrO diffraction peaks due to the low doping concentration, the EDS elemental mapping conclusively demonstrated successful strontium incorporation in the scaffold architecture. The mechanical strength of the scaffolds is illustrated in Figure 2D and E. Notably, all Sr-doped TCP scaffolds exhibited higher elastic modulus compared to the pure TCP group. Specifically, the 5Sr-TCP group demonstrated a compressive strength of 1.44 MPa, while both 2.5Sr-TCP and 10Sr-TCP scaffolds showed comparable strength values, all surpassing those of undoped TCP scaffolds. *In vitro* degradation assessment of the TCP and Sr-doped TCP scaffolds in SBF over 3 weeks revealed distinct behaviors (Figure 2F). Pure TCP maintained structural stability with negligible mass change, while 2.5Sr-TCP and 5Sr-TCP showed progressive mass gains indicative of surface deposition. Conversely, 10Sr-TCP exhibited significant mass loss. Synchronized ion release profiles (Figure 2G and H) demonstrated accelerated Ca^2+^ dissolution in 10Sr-TCP, alongside Sr^2+^ release intensities scaling with doping concentration. Notably, 10Sr-TCP displayed a kinetic transition after Day 5, characterized by a plateau in Ca^2+^/Sr^2+^ release rates and confirming optimized dual-ion elution.

**Table 3.** Elemental composition of Sr-doped β-TCP.

Sample	Elemental composition (wt%)		
	P	Ca	Sr
TCP	42.53	57.47	0
2.5Sr-TCP	39.47	54.04	6.49
5Sr-TCP	31.77	56.8	11.43
10Sr-TCP	30.88	52.51	16.61

### Biocompatibility assessments of Sr-doped TCP scaffolds

**Figure 3.**
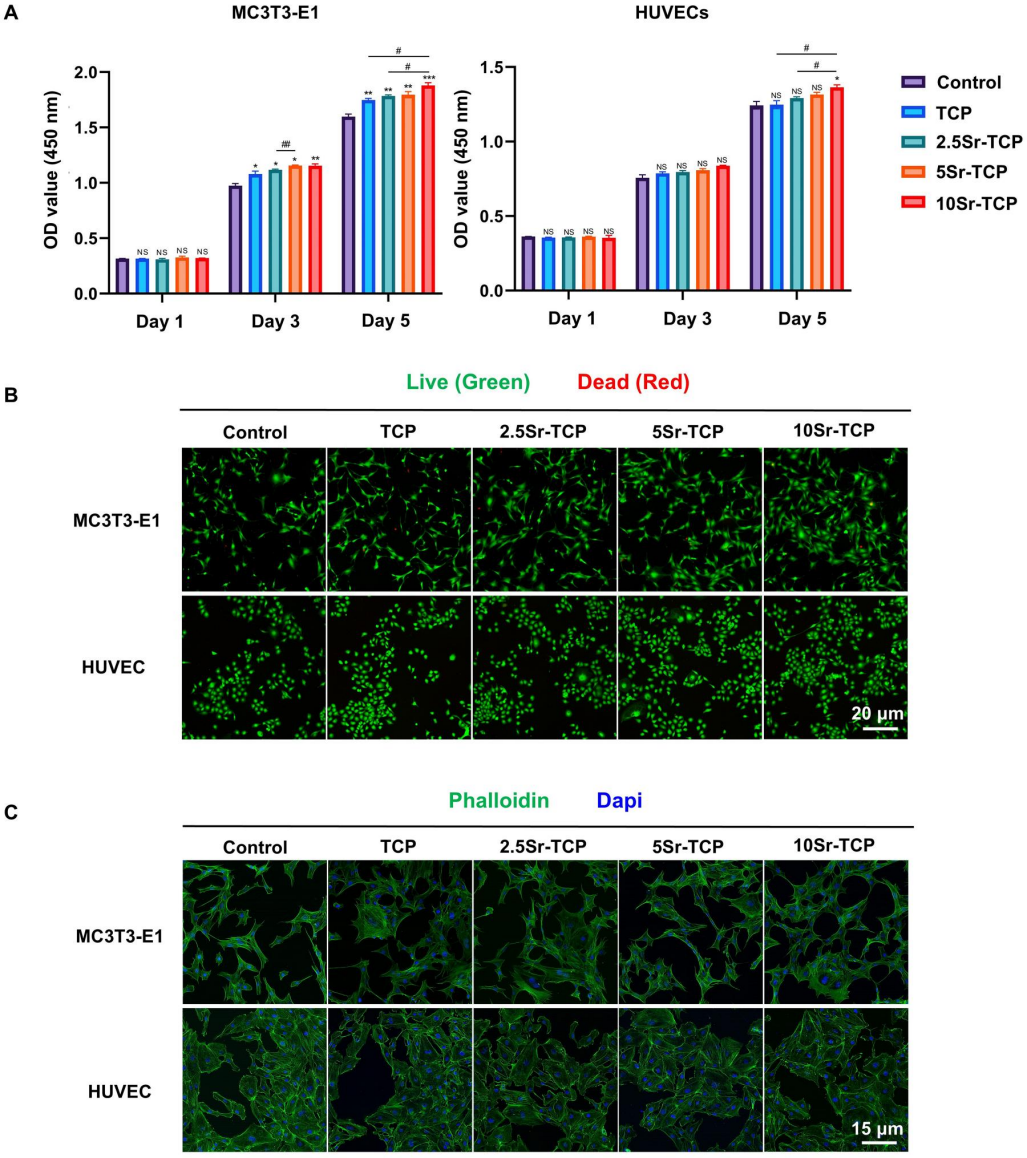
Biocompatibility evaluation of TCP and Sr-TCP scaffolds. (**A**) CCK-8 assays of MC3T3-E1 cells and HUVECs cultured with TCP extract (TCP), 2.5Sr-TCP extract (2.5Sr-TCP), 5Sr-TCP extract (5Sr-TCP), 10Sr-TCP extract (10Sr-TCP) and α-MEM medium (control) at 1, 3 and 5 days. (**B**) Live/dead staining images of MC3T3-E1 cells and HUVECs after 3 days of coculture. (**C**) Cytoskeleton and nucleus staining of MC3T3-E1 cells and HUVECs after 72 h. (*n* = 3; ^NS^P > 0.05, *P < 0.05, **P < 0.01, ***P < 0.001, ****P < 0.0001 versus control group; ^#^*P* < 0.05, ^##^*P* < 0.01, ^###^*P* < 0.001, ^####^*P* < 0.0001 among experimental groups).

As depicted in Figure 3A, MC3T3-E1 cells exhibited progressive proliferation with extended culture time, with all TCP scaffolds enhancing cell growth from Day 3 onward. Notably, Sr-incorporated groups demonstrated increased OD values correlating with higher Sr concentrations, indicating that Sr incorporation significantly boosted cellular growth activity. Similarly, HUVECs showed a time-dependent increase in OD values across all groups, underscoring the favorable cytocompatibility of Sr-TCP scaffolds. Live/Dead cell staining further corroborated these findings (Figure 3B). Both MC3T3-E1 cells and HUVECs exhibited high viability with negligible cell death observed on Day 3. Additionally, Phalloidin/Dapi staining after 48 h of incubation revealed well-spread cells with intact nuclei and cytoskeletons in all groups, devoid of any signs of curling, shrinkage or fragmentation (Figure 3C). Collectively, these results affirm that Sr-doped TCP scaffolds exhibit exceptional biocompatibility, effectively supporting cell proliferation and spreading.

### Cell adhesion and migration assessments of Sr-doped TCP scaffolds in MC3T3-E1 cells

**Figure 4.**
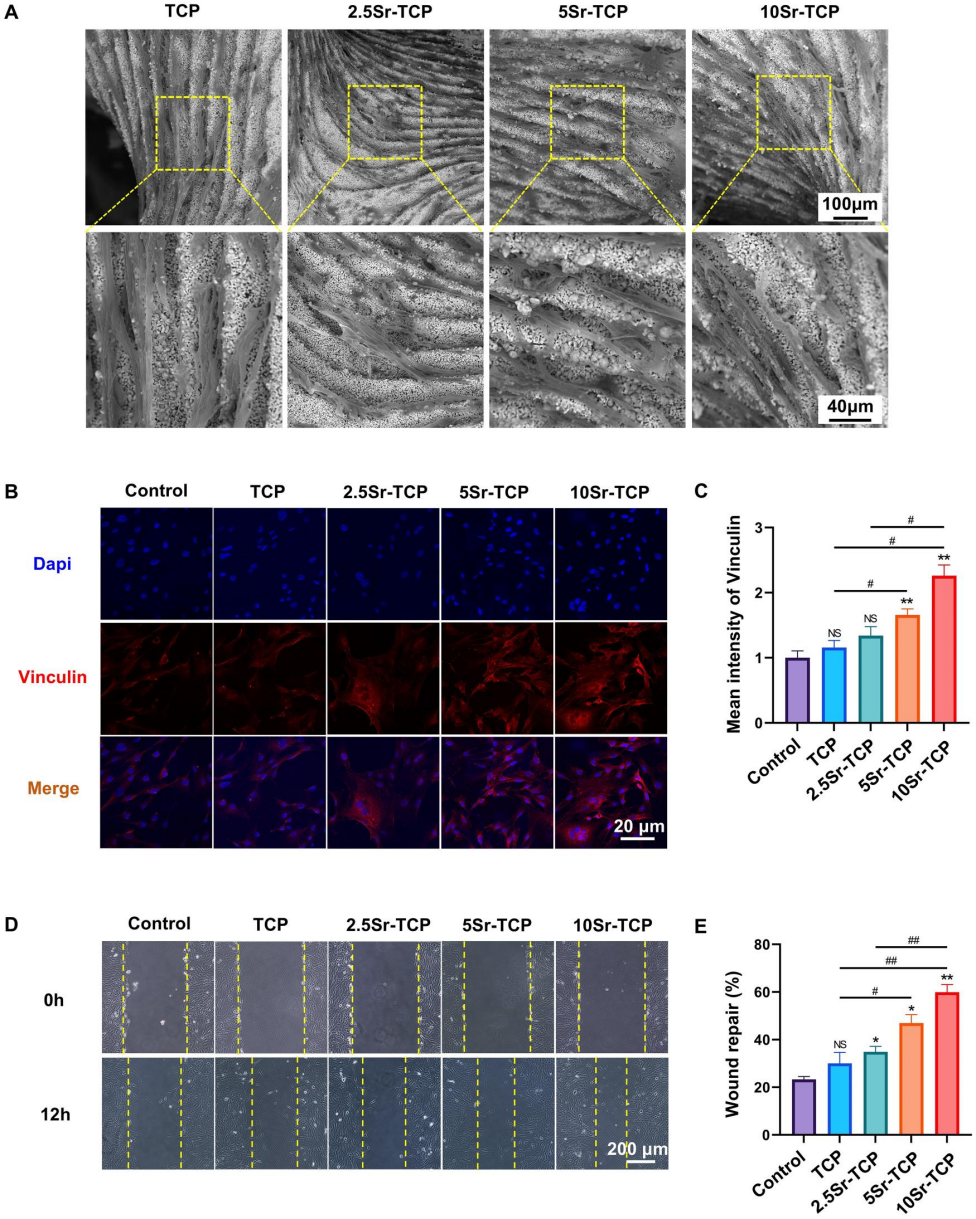
Evaluation of cell adhesion and migration on TCP and Sr-TCP scaffolds in MC3T3-E1 cells. (**A**) SEM images of MC3T3-E1 cells adhered to TCP and Sr-TCP scaffolds after 48 h. (**B**) Immunofluorescent staining and (**C**) quantitative analysis of vinculin expression in MC3T3-E1 cells treated with TCP extract (TCP), 2.5Sr-TCP extract (2.5Sr-TCP), 5Sr-TCP extract (5Sr-TCP), 10Sr-TCP extract (10Sr-TCP) and α-MEM medium (control) after 48 h. (**D**) Microscopy images and (**E**) quantitative analysis of MC3T3-E1 cell migration in various extracts and α-MEM medium during scratch wound healing assays. (*n* = 3; ^NS^P > 0.05, ^*^P < 0.05, ^**^P < 0.01, ^***^P < 0.001, ^****^P < 0.0001 versus control group; ^#^*P* < 0.05, ^##^*P* < 0.01, ^###^*P* < 0.001, ^####^*P* < 0.0001 among experimental groups).

As depicted in Figure 4A, MC3T3-E1 cells were cultured on both TCP and Sr-doped TCP scaffolds. Cells in all groups exhibited a spreading morphology with extended pseudopodia, demonstrating robust adhesion to the scaffolds. Notably, cells adopted a spindle-like shape, aligning with the layered ravines formed during 3D printing. Additionally, vinculin, a key focal adhesion marker, was assessed via immunofluorescence staining (Figure 4B and C). Vinculin expression increased with higher Sr concentrations in TCP scaffolds, peaking in the 10Sr-TCP group. As demonstrated in scratch wound assays, MC3T3-E1 cells in Sr-doped TCP groups exhibited significant, concentration-dependent wound closure, whereas no notable difference was observed between the control and TCP groups (Figure 4D and E). These findings suggest that Sr-doped TCP scaffolds significantly enhance cell adhesion and migration, with Sr incorporation promoting these effects, thereby potentially supporting osteogenesis.

### Evaluation of osteogenic differentiation of Sr-doped TCP scaffolds in MC3T3-E1 cells

**Figure 5.**
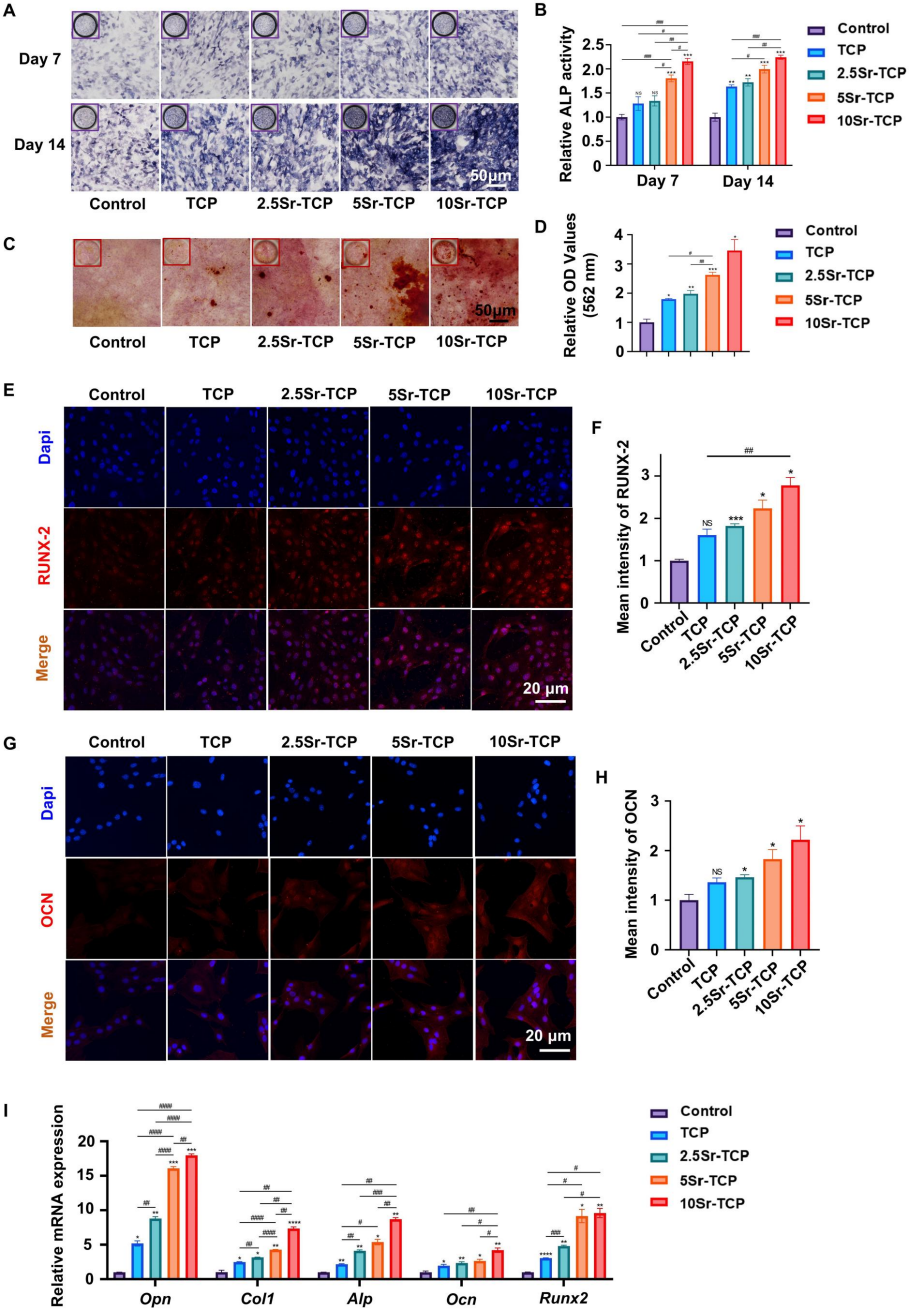
*In vitro* osteogenic differentiation of TCP and Sr-TCP scaffolds in MC3T3-E1 cells. (**A**) ALP staining and (**B**) relative ALP activity of MC3T3-E1 cells cultured with TCP extract (TCP), 2.5Sr-TCP extract (2.5Sr-TCP), 5Sr-TCP extract (5Sr-TCP), 10Sr-TCP extract (10Sr-TCP) and α-MEM medium (control) at 7 and 14 days. (**C**) ARS staining and (**D**) semi-quantitative analysis of mineralized nodules in each group at 21 days. (**E**) Immunofluorescent images and (**F**) quantitative analysis of RUNX2 expression in MC3T3-E1 cells at 7 days. (**G**) Immunofluorescent images and (**H**) quantitative analysis of OCN expression at 14 days. (**I**) Relative mRNA expression of osteogenic genes (*Opn, Col1, Alp, Ocn, Runx2*) in MC3T3-E1 cells at 7 days. (*n* = 3; ^NS^P > 0.05, ^*^P < 0.05, ^**^P < 0.01, ^***^P < 0.001, ^****^P < 0.0001 versus control group; ^#^*P* < 0.05, ^##^*P* < 0.01, ^###^*P* < 0.001, ^####^*P* < 0.0001 among experimental groups).

The impact of Sr-doped TCP scaffolds on osteogenic differentiation of MC3T3-E1 cells was systematically evaluated. ALP staining revealed enhanced osteogenic activity with increasing Sr concentrations at both Day 7 and Day 14 (Figure 5A). Quantitative analysis demonstrated that 5Sr-TCP and 10Sr-TCP significantly boosted ALP expression on Day 7, while TCP and 2.5Sr-TCP showed comparable results to the control (Figure 5B). By Day 14, all Sr-doped scaffolds exhibited dose-dependent ALP activity enhancement, with 10Sr-TCP achieving peak performance. ARS staining (Figure 5C) and subsequent semi-quantitative analysis (Figure 5D) confirmed superior mineralization in 10Sr-TCP scaffolds by Day 21. Immunofluorescence analysis of osteogenic markers revealed concentration-dependent improvements in both nuclear RUNX2 expression (Figure 5E and F) and cytoplasmic OCN localization (Figure 5G and H), with 10Sr-TCP demonstrating maximal expression. Additionally, qPCR analysis of osteogenic genes (Supplementary Figure S1 and Figure 5I) further validated these findings, showing dose-dependent upregulation of *Opn*, *Col1*, *Alp*, *Ocn* and *Runx2* in Sr-TCP groups. Collectively, these results demonstrate that Sr incorporation into TCP scaffolds significantly enhances osteogenic differentiation of MC3T3-E1 cells through a dose-dependent mechanism, as evidenced at genetic, protein and cellular levels.

### Evaluation of angiogenic activity of Sr-doped TCP scaffolds in HUVECs

**Figure 6.**
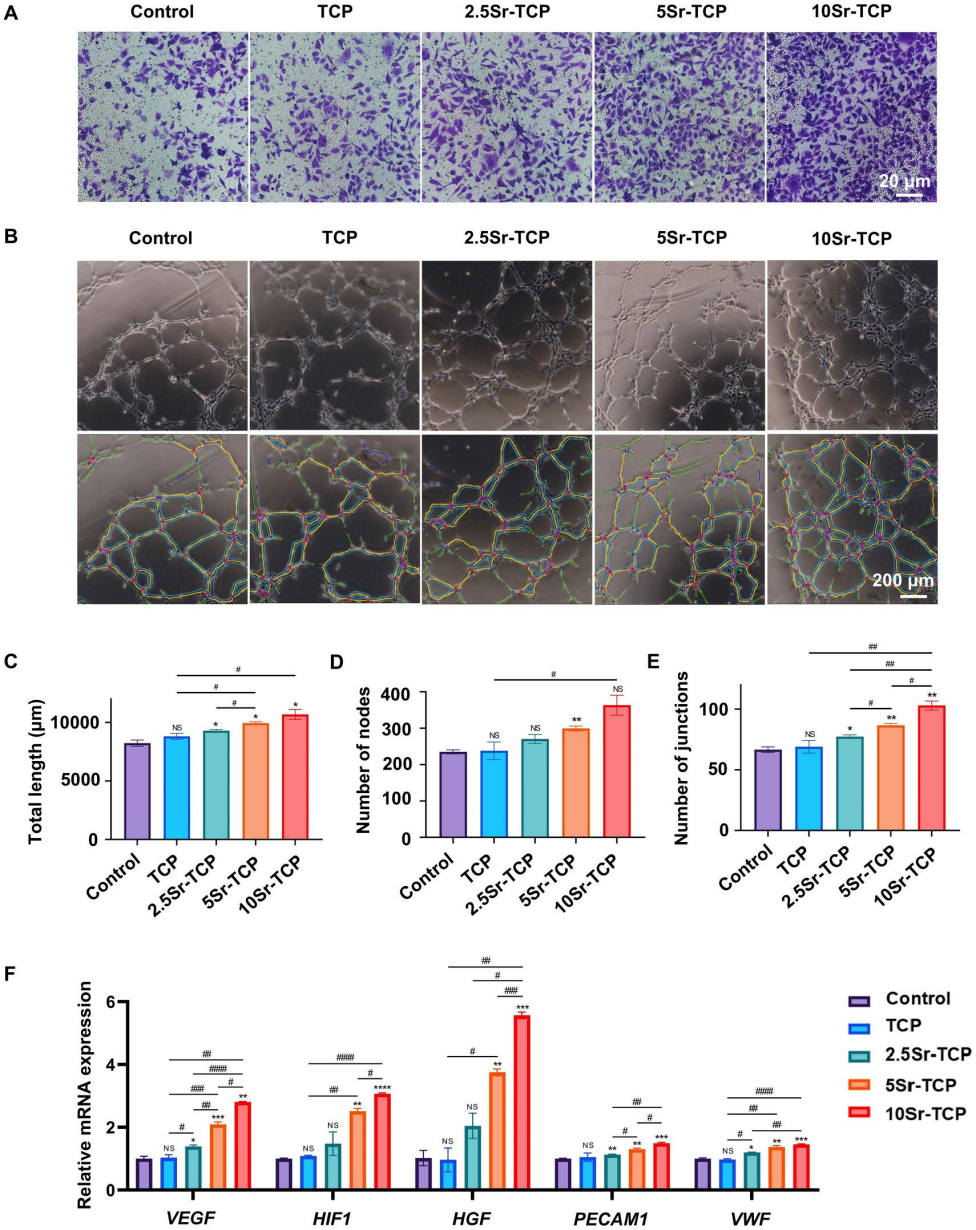
*In vitro* angiogenic activity of TCP and Sr-TCP scaffolds in HUVECs. (**A**) Microscopic images of migrated HUVECs following 24-h treatment with TCP extract (TCP), 2.5Sr-TCP extract (2.5Sr-TCP), 5Sr-TCP extract (5Sr-TCP), 10Sr-TCP extract (10Sr-TCP) and ECM medium (control) in transwell assays. (**B**) Images of tube formation and quantitative analyses of (**C**) total length, (**D**) number of nodes and (**E**) number of junctions in HUVECs after 4 h. (**F**) Relative mRNA expression of angiogenesis-related genes (*VEGF, HIF1, HGF, PECAM1, VWF*) in HUVECs after 3 days. (*n* = 3; ^NS^P > 0.05, *P < 0.05, **P < 0.01, ***P < 0.001, ****P < 0.0001 versus control group; ^#^*P* < 0.05, ^##^*P* < 0.01, ^###^*P* < 0.001, ^####^*P* < 0.0001 among experimental groups).

Transwell migration assays revealed that Sr-doped TCP groups significantly enhanced HUVECs migration in a concentration-dependent manner (Figure 6A). Furthermore, the tube formation capability of HUVECs co-cultured with various sample extracts on Matrigel was assessed. As depicted in Figure 6B, HUVECs in Sr-doped TCP groups formed distinct tubular structures, whereas the control and TCP groups exhibited predominantly discontinuous tubular walls after 4 h of incubation. Statistical analysis confirmed the superior angiogenic properties of Sr-doped TCP scaffolds, with the 10Sr-TCP group showing significantly higher values for total tube length, number of nodes and junctions compared to other groups (Figure 6C–E). Additionally, HUVECs cultured in Sr-doped TCP extracts displayed markedly higher expression levels of angiogenesis-related genes (*VEGF, HIF1, HGF, PECAM1, VWF)* in qPCR assays (Figure 6F). Specifically, the 10Sr-TCP group achieved the highest expression of target genes after 3 days of co-culture. These *in vitro* angiogenesis assessments demonstrated that Sr incorporation significantly enhanced the angiogenic properties of TCP scaffolds in a dose-dependent manner, with the 10Sr-TCP scaffolds exhibiting the most potent pro-angiogenic effects.

### *In vivo* vascularized bone regeneration of Sr-doped TCP scaffolds

**Figure 7.**
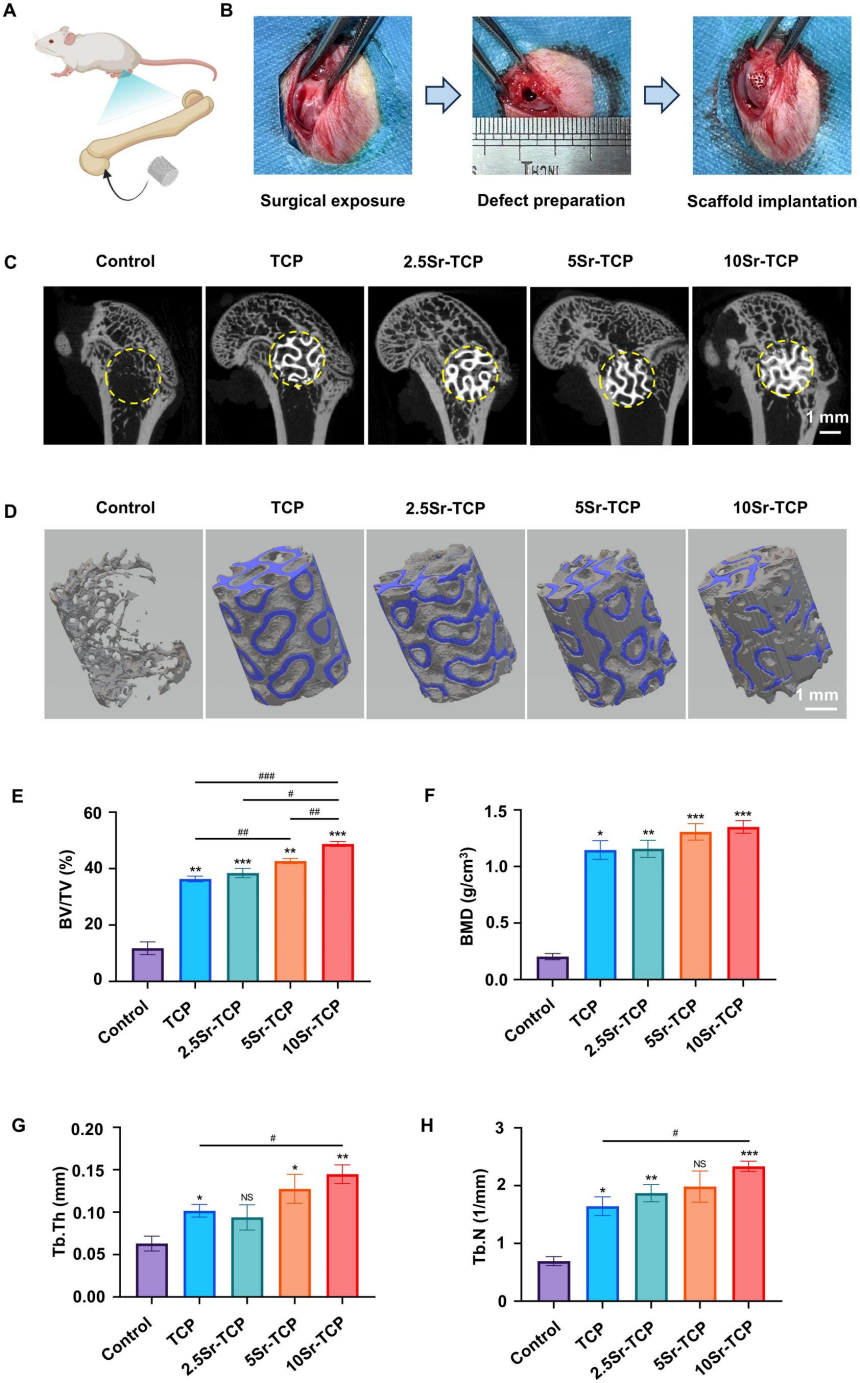
Micro-CT analysis of the impact of TCP and Sr-TCP scaffolds on *in vivo* bone regeneration. (**A**) Schematic illustration and (**B**) macroscopic images of the surgical procedure. (**C**) Sagittal views and (**D**) 3D reconstructions of the rat femoral condylar defects treated with TCP, 2.5Sr-TCP, 5Sr-TCP and 10Sr-TCP scaffolds after 8 weeks of implantation. (**E**) BV/TV, (**F**) BMD, (**G**) Tb.Th and (**H**) Tb.N of the femoral condylar defects quantified through Micro-CT analysis (*n* = 3; ^NS^P > 0.05, *P < 0.05, **P < 0.01, ***P < 0.001, ****P < 0.0001 versus control group; ^#^*P* < 0.05, ^##^*P* < 0.01, ^###^*P* < 0.001, ^####^*P* < 0.0001 among experimental groups).

To evaluate the enhanced capacity of vascularized bone regeneration *in vivo*, a rat femoral condylar defect model was established. Figure 7A outlines the surgical procedure, with intraoperative images presented in Figure 7B. Sagittal X-ray CT images (Supplementary Figure S2A) revealed minimal new bone formation in the control group, while significantly more new bone was observed in other groups at 4 weeks. Notably, the 10Sr-TCP group exhibited the largest new bone coverage area compared to groups with lower Sr concentrations. After 8 weeks, new bone formation increased, with nearly half of the defect area covered in the 10Sr-TCP group (Figure 7C). Additionally, 3D reconstruction images (Supplementary Figure S2B and  Figure 7D) confirmed that Sr-doped TCP scaffolds promoted greater new bone formation (grey area) in a concentration-dependent manner. Quantitative analysis of BV/TV values showed a significant increase in the TCP (32.21 ± 1.68%; 36.31 ± 1.00%), 2.5Sr-TCP (32.10 ± 2.71%; 38.44 ± 1.63%), 5Sr-TCP (37.75 ± 2.00%; 42.69 ± 0.88%) and 10Sr-TCP (41.34 ± 1.47%; 48.71 ± 0.84%) groups compared to the control group (6.66 ± 0.83%; 11.77 ± 2.25%) at 4 and 8 weeks (Supplementary Figure S2C and  Figure 7E), respectively, indicating accelerated bone regeneration. Furthermore, Sr-doped TCP groups demonstrated higher BMD, Tb.Th and Tb.N values in a dose-dependent manner, with the 10Sr-TCP group achieving the highest values at 4 and 8 weeks (Supplementary Figure S2D–F and Figure 7F–H). Overall, radiographic evaluation confirmed the superior osteogenic capacity of 10Sr-TCP scaffolds in the rat femoral condylar defect model, consistent with *in vitro* findings.

**Figure 8.**
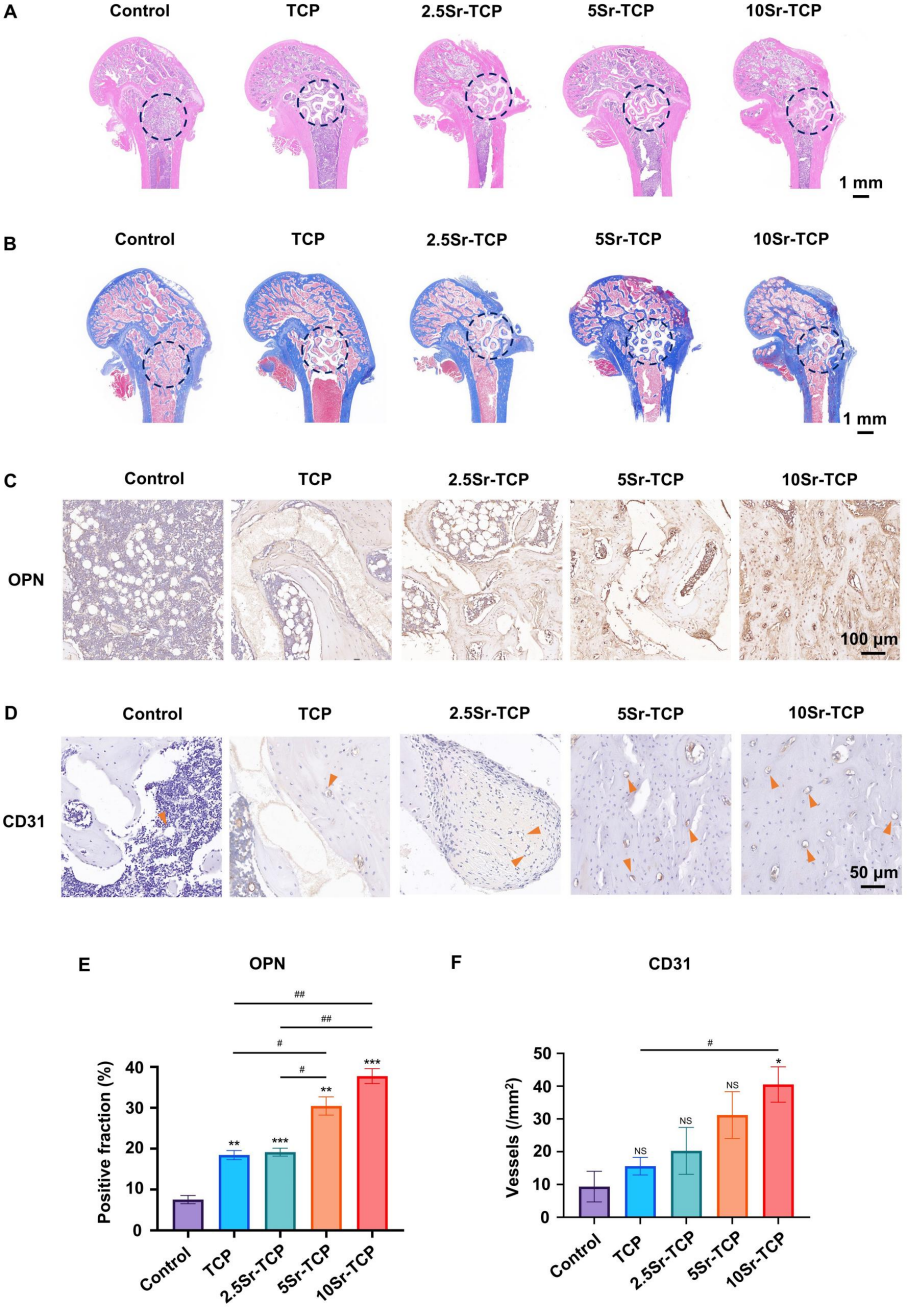
Histological evaluation of bone regeneration *in vivo*. (**A**) H&E and (**B**) MT staining images of the defect areas in control, TCP, 2.5Sr-TCP, 5Sr-TCP and 10Sr-TCP groups at 8 weeks post-implantation. (**C**) Immunohistochemical staining images of OPN, with (**E**) semi-quantitative analysis across groups. (**D**) Immunohistochemical staining images of CD31, with (**F**) semi-quantitative analysis of the vessels number across groups. Arrows point to the vessels. (*n* = 3; ^NS^P > 0.05, ^*^P < 0.05, ^**^P < 0.01, ^***^P < 0.001 versus control group; ^#^*P* < 0.05, ^##^*P* < 0.01 among experimental groups).

As shown in the H&E staining images (Supplementary Figure S3A and  Figure 8A), fibrous connective tissues predominated in the defect areas of the control group, with minimal new bone formation. In contrast, both TCP and Sr-TCP groups exhibited increased bone regeneration as the Sr concentration progressively increased. Notably, the 10Sr-TCP group demonstrated the most significant bone regeneration after 8 weeks of implantation, with a substantial amount of new bone integrating into the scaffolds, resulting in nearly 50% healing. Additionally, MT staining results illustrated that the Sr-TCP groups displayed superior collagen fiber bundles and ossified tissues in the defect areas in a dose-dependent manner (Supplementary Figure S3B and  Figure 8B). As illustrated in Supplementary Figure S3C and  Figure 8C, the expression intensity of OPN was enhanced in the Sr-TCP groups with increasing Sr concentrations. Semi-quantitative analysis of the immunohistochemical staining of OPN in Supplementary Figure S3E and  Figure 8E revealed that the 10Sr-TCP scaffold showed the highest fraction of positive cells after 4 and 8 weeks of implantation. Moreover, the angiogenic factor CD31 also exhibited the highest positive cell fraction in the 10Sr-TCP scaffold (Supplementary Figure S3D and  Figure 8D), as confirmed by quantitative analysis (Supplementary Figure S3F and  Figure 8F). In conclusion, incorporating Sr into TCP scaffolds, particularly the 10Sr-TCP scaffold, synergistically enhances angiogenesis and osteogenesis, facilitating vascularized bone regeneration *in vivo*.

## Discussion

Critical-size bone defects in the oral and maxillofacial region remain significant clinical challenges. While β-TCP scaffolds are recognized as promising biomaterials for bone tissue engineering, their limited vascularized osteogenesis hampers widespread application [29]. Recent studies have highlighted the critical roles of bioactive dopants and porous structures in bone regeneration [30]. To address the unmet need for scaffolds that simultaneously optimize structural functionality and safe bioactivity, we employed DLP-based 3D printing to fabricate Sr-doped TCP bioceramic TPMS scaffolds with varying Sr concentrations, establishing the first comprehensive analysis of Sr dosage effects within a TPMS framework for dual osteogenic/angiogenic enhancement. Our findings reveal that the 10Sr-TCP scaffold exhibits favorable compressive stress and significantly enhances new bone regeneration and vessel formation both *in vitro* and *in vivo*, positioning it as a promising biomaterial for bone defect restoration.

### Design and characterization of Sr-doped TCP scaffolds

Since the 1980s, calcium phosphate-based materials have been widely adopted in bone tissue engineering owing to their excellent biocompatibility, bioactivity and adjustable biodegradation [31]. Recently, the TPMS structure, characterized by interconnected pores, extensive surface area and scalable topology, has garnered significant attention [32]. The unique TPMS architecture promotes cell recruitment, differentiation and vascular ingrowth, enabling enhanced vascularized bone regeneration at reduced Sr doses, thereby minimizing potential side effects. Furthermore, Sr plays a crucial role in osteogenesis and angiogenesis within specific concentration ranges [33]. Leveraging these properties, Sr-doped TCP bioceramic TPMS scaffolds were developed using DLP-based 3D printing technology, which offers exceptional advantages in dimensional precision and internal microstructure control. The Sr doping concentrations (0, 2.5, 5.0 and 10.0 mol%) were selected based on three key rationales. First, systematic studies indicate that Sr incorporation in calcium phosphate systems exceeding 10 mol% leads to plateauing effects on osteogenic markers without proportional improvements in bone regeneration efficiency [34]. Second, to mitigate risks associated with supra-physiological Sr doses [35, 36], we adhered to the 10 mol% threshold, balancing bioactivity and biocompatibility. Most critically, a core innovation of this work is the integration of Sr doping with a TPMS architecture to achieve enhanced bioactivity at reduced Sr doses, rather than exploring higher Sr concentrations. Therefore, to determine the optimal strontium concentration in β-TCP scaffolds featuring TPMS structures for enhanced bone regeneration, the scaffolds were systematically doped with Sr at concentrations of 0, 2.5, 5.0 and 10.0 mol%.

In clinical practice, the Sr-TCP scaffold demonstrates significant potential for oral and maxillofacial applications, where its biomechanical compatibility (1.13–1.44 MPa) matches the native trabecular bone environment (1.37–4.83 MPa) [37]. The enhanced mechanical performance, despite the high porosity of the TPMS structure, stems primarily from its continuously curved surfaces, which efficiently absorb mechanical energy [38]. This has led to the growing application of TPMS-structured scaffolds in weight-bearing bone restoration in recent years [39]. While their compressive stress remains lower than that of solid scaffolds, the porous architecture of TPMS structures effectively mimics the heterogeneity of damaged tissues and provides ample space for bone ingrowth.

Regarding *in vitro* degradation properties, the TCP, 2.5Sr-TCP and 5Sr-TCP scaffolds demonstrated predominant mineralization behavior during 3-week SBF immersion, as evidenced by their mass increase rate. This phenomenon primarily stemmed from surface mineral deposition, a process significantly enhanced by Sr^2+^ doping [40]. Notably, 2.5Sr-TCP exhibited the most pronounced mineralization effect. In contrast, 5Sr-TCP showed attenuated mass gain with fluctuations, suggesting nearly balanced mineralization-degradation kinetics due to partial degradation offset. Strikingly, 10Sr-TCP displayed degradation-dominated behavior, attributable to accelerated Sr^2+^/Ca^2+^ release. Importantly, while promoting early osteogenesis, scaffold degradation simultaneously creates progressively expanding space for new bone ingrowth. Additionally, the ion release study reveals the distinctive synergistic effects and biological significance of dual Ca^2+^/Sr^2+^ ions in Sr-doped TCP scaffolds. Remarkably, all Sr-doped groups showed enhanced Ca^2+^ release despite lower calcium content—a phenomenon resulting from the incorporation of strontium [41, 42]. The replacement of Ca^2+^ (1.00 Å) with larger Sr^2+^ ions (1.18 Å) expands ionic spacing in the β-TCP crystal structure, accelerating dissolution kinetics. Importantly, the coordinated release of both ions creates an optimal bioactive environment for bone regeneration. The Sr^2+^ release concentrations observed in this study align with the established therapeutic safety window of 3–12 mM reported for bone regeneration applications [43]. Notably, the 10Sr-TCP group exhibited particularly favorable kinetics, reaching a stable release plateau after 5 days—this controlled release profile minimizes cytotoxicity risks while sustaining therapeutic effects through maintained ion concentrations.

### *In vitro* studies of osteogenic adhesion, migration and differentiation

As Sr concentration increased, MC3T3-E1 cells cultured on 10Sr-TCP scaffolds displayed an outstretched morphology, accompanied by significantly enhanced vinculin expression and improved migration activity. Cell behavior at the material-tissue interface is primarily governed by two key factors: topographical characteristics and biologically active dopants within the biomaterial [44]. Notably, TPMS scaffolds have demonstrated superior surface area-to-volume ratios and enhanced permeability, which facilitate cell adhesion and migration [45]. Yang *et al*. further revealed that the intricate reorganization of stress fibers and focal adhesions in TPMS structures may trigger a cascade of cellular responses during osteogenesis and angiogenesis [14]. Moreover, numerous studies have established that Sr incorporation fosters a favorable microenvironment for cell attachment [46, 47], aligning with our observations. Specifically, Zhou *et al*. demonstrated that Sr treatment enhanced mesenchymal stem cell adhesion and migration, correlating with increased CDH2 expression—a classical cadherin family member [48]. These findings collectively suggest that the enhanced cell adhesion and migration observed in 10Sr-TCP scaffolds promote favorable host-implant interactions, facilitating osteoblast recruitment from surrounding tissues for bone regeneration.

The osteogenic potential of Sr-doped TCP scaffolds was comprehensively validated through cellular, protein and genetic analyses, with 10 mol% Sr incorporation emerging as the optimal formulation for bone regeneration. During osteogenic differentiation, ALP serves as an early-stage marker [49], while mineralized calcium nodules indicate the terminal phase [50]. The process is regulated by RUNX2, a nuclear transcription factor crucial for osteogenic differentiation [51] and OCN, a non-collagenous bone matrix protein essential for cellular maturation and mineralization [52]. Our findings demonstrated that Sr-doped TCP scaffolds significantly enhanced osteogenic differentiation and extracellular matrix mineralization in a dose-dependent manner. This enhancement was mediated through the upregulation of osteogenesis-related genes and increased expression of RUNX2 and OCN proteins. The observed *in vitro* osteogenic activity likely resulted from the synergistic release of Sr^2+^ and Ca^2+^ ions, consistent with previous studies on Sr-Ca co-doped scaffold materials [53, 54]. Strontium ions, sharing a similar ionic radius with Ca^2+^, primarily mediate osteogenesis through shared signaling pathways such as Wnt/β-catenin and PI3K/AKT/mTOR [55, 56], where Sr^2+^ binds to calcium-sensing receptors (CaSR) to amplify osteoblast differentiation and bone formation. Beyond mimicking Ca^2+^, Sr^2+^ uniquely modulates mitochondrial function in macrophages by activating the PI3K/AKT/mTOR pathway, which enhances oxidative phosphorylation and reduces reactive oxygen species (ROS) production, thereby promoting a metabolic shift toward the pro-regenerative M2 macrophage phenotype [57]. This immunomodulatory effect synergizes with the direct osteogenic actions of Sr^2+^, including the upregulation of type I collagen secretion and extracellular matrix mineralization [58], which collectively establish a favorable microenvironment for bone remodeling. By integrating these dual roles—leveraging shared Ca^2+^-dependent pathways while introducing Sr^2+^-specific mitochondrial and immunoregulatory mechanisms—the scaffold achieves enhanced osteointegration and vascularized bone regeneration. While excessive Sr exposure can inhibit bone formation, the optimal concentration range remains debated due to variations in experimental conditions and cell types [35, 36]. Notably, our study identified 10 mol% Sr-doped TCP scaffolds as demonstrating superior osteogenic properties among all tested formulations.

### *In vitro* studies of angiogenic activity

Bone tissue, a highly vascularized structure, relies on robust vessel formation as a critical prerequisite for bone regeneration. The dense vascular network within bone defects facilitates mesenchymal stem cell recruitment and differentiation [59]. Our analysis focused on three key indicators of *in vitro* angiogenic activity: HUVECs proliferation, migration and tube formation capacity. Among angiogenic regulators, *VEGF* stands out as a pivotal molecule in blood vessel invasion [60], while *HIF1* orchestrates osteogenic differentiation through the VEGF/AKT/mTOR signaling pathway, effectively coupling osteogenesis with angiogenesis [61, 62]. Furthermore, *HGF*, *PECAM1* and *VWF* significantly contribute to bone marrow erythropoiesis, cellular interactions and endothelial function [63, 64], all of which are intricately linked to vascular development. Notably, our 10Sr-TCP scaffolds demonstrated superior angiogenic induction potential, significantly enhancing HUVECs proliferation, migration and tube formation through the upregulation of multiple angiogenesis-related genes.

Calcium phosphate has been widely recognized for its limited angiogenic properties, which significantly hinder its clinical utility. To address this, incorporating bioactive dopants has emerged as a promising strategy to enhance vascularization during bone regeneration. Among these, strontium has garnered considerable attention due to its well-documented pro-angiogenic effects [65, 66]. Numerous studies have demonstrated that Sr-modified calcium phosphate scaffolds significantly improve cell migration and tube formation [67], consistent with our research. Notably, Wu *et al*. developed a strontium-calcium phosphate hybrid cement that substantially improved neovascularization in a rat subcutaneous implantation model [68]. Despite these advances, the precise mechanisms underlying angiogenic effects of strontium remain elusive. While Sr is known to directly influence cellular behaviors such as proliferation, interaction and migration [69], emerging evidence suggests it may also modulate angiogenesis-related factors through paracrine signaling [70]. Furthermore, Cheng *et al*. revealed that Sr promoted neovascularization by activating the ERK1/2 signaling pathway [71]. Our *in vitro* experiments demonstrated that the 10Sr-TCP scaffolds, which exhibited a concentration-dependent enhancement of tube formation, may offer significant advantages in promoting bone regeneration, particularly for large bone defects, due to the critical role of vascularized grafts in tissue defect repair.

### *In vivo* studies of vascularized bone regeneration

Sr-doped TCP scaffolds markedly accelerated bone regeneration in a rat femoral condylar defect model, while the control group exhibited delayed bone healing, primarily progressing from the periphery. Notably, all TCP groups demonstrated robust new bone formation, both within the porous TPMS structure and along the scaffold edges. The emergence of secondary osteogenesis centers within the defect was primarily due to the TPMS-structured scaffolds, whose interconnected pores facilitated cell recruitment, attachment and capillary ingrowth. Moreover, the release of bioactive Sr^2+^ and Ca^2+^ ions from the scaffolds markedly accelerated the healing process, yielding superior outcomes compared to the control group. Notably, the 10Sr-TCP group exhibited the highest BMD and BV/TV values at all-time points, likely due to the optimal local concentrations of Sr^2+^ and Ca^2+^ ions. OPN, a key regulator of bone formation, modulates osteoblast adhesion and matrix mineralization [72], while CD31 serves as a specific biomarker for angiogenesis and endothelial differentiation. Higher OPN expression was detected in Sr-doped TCP groups, correlating with increased vascularization marked by CD31. Importantly, studies have highlighted a synergistic relationship between angiogenesis and osteogenesis [73, 74]. Endothelial cell-derived extracellular factors promote osteogenic differentiation and inhibit osteoblast apoptosis [75], while mesenchymal stem cell-derived exosomes regulate angiogenesis through the secretion of angiogenic molecules [76]. This interplay between angiogenesis and osteogenesis is crucial for accelerating new bone formation. Although this study provides a preliminary evaluation of *in vivo* osteogenic and angiogenic performance, more comprehensive assessments, including laser Doppler imaging, fluorescent microsphere perfusion and tetracycline labeling, are warranted to further elucidate the mechanistic and functional outcomes.

In summary, both *in vitro* and *in vivo* results demonstrated that Sr-incorporated TPMS-structured TCP scaffolds promoted vascularized bone regeneration in a concentration-dependent manner, with 10Sr-TCP scaffolds showing the most favorable outcomes. Collectively, our work introduces a transformative “structure-enhanced bioactivity” paradigm by synergizing TPMS topology with low-dose Sr doping, resolving the dose-toxicity trade-off that limits conventional bioceramics. Three key innovations distinguish our work from prior research: (1) We resolve the long-standing dichotomy between structural integrity and biofunctional efficacy by synergizing TPMS topological optimization with precision Sr doping. Unlike conventional single-parameter approaches, our integrated strategy leverages TPMS hyperboloidal geometry to confer exceptional mechanical strength (1.44 MPa at 80% porosity) while enabling controlled Sr release kinetics that amplify bioactivity at minimal doses. (2) We establish a new safety-efficacy paradigm for bioactive ions by demonstrating that 10 mol% Sr, substantially lower than therapeutic thresholds in conventional scaffolds [26, 77], induces maximal osteogenic/angiogenic enhancement when delivered through a TPMS architecture. This dose reduction directly mitigates Sr accumulation risks, overcoming a critical barrier to clinical translation. (3) We systematically elucidate the dose-dependent biological effects of Sr within a TPMS framework, establishing clear correlations between Sr concentrations and cellular responses, molecular mechanisms and functional outcomes. Our findings establish that 10 mol% Sr-TCP scaffolds optimally balance structural integrity, bioactivity and clinical safety, providing a blueprint for next-generation patient-specific implants where biomimetic design amplifies low-dose therapeutic efficacy.

However, this study has limitations. First, the role of inflammatory responses in bone regeneration warrants further investigation, particularly regarding the effects of Sr-doped TCP scaffolds on inflammatory cytokine secretion and macrophage polarization post-implantation. Second, while Sr incorporation significantly enhanced angiogenesis and osteogenesis, the underlying mechanisms of the synergistic effects between Sr^2+^ and Ca^2+^ ions and the associated signaling pathways remain to be elucidated. Finally, long-term *in vivo* studies are needed to provide robust evidence for clinical translation.

## Conclusions

In summary, we successfully fabricated Sr-doped TCP bioceramic TPMS scaffolds using DLP-based 3D printing, identifying 10 mol% Sr as the optimal concentration for enhancing angiogenic and osteogenic properties. The biomimetic TPMS structure uniquely provided exceptional mechanical strength at high porosity (80%), overcoming a key limitation in scaffold design. Crucially, this study demonstrates that integrating the TPMS architecture with precision Sr-doping creates a synergistic effect, enabling potent bioactivity at significantly reduced Sr concentrations, thereby mitigating potential toxicity risks. *In vitro* experiments revealed that Sr-doped TCP scaffolds not only promoted osteogenic differentiation and mineralization in MC3T3-E1 cells in a concentration-dependent manner, but also enhanced the proliferation, migration and tube formation capabilities of HUVECs. Furthermore, *in vivo*, the TPMS-structured Sr-TCP scaffolds significantly accelerated vascularized osteogenesis, leading to markedly improved bone regeneration in rat femoral condylar defects. Collectively, these findings resolve the critical challenge of achieving effective vascularized regeneration with safe ion doses by establishing a novel “structure-enhanced bioactivity” paradigm. This synergistic design strategy, leveraging biomimetic topology to amplify low-dose ion efficacy, not only advances the scientific understanding of scaffold-cell interactions but also provides a transformative and clinically translatable platform for safe and efficient bone tissue engineering.

## Supplementary Material

rbaf080_Supplementary_Data
