# Supplementary material for: Three-dimensional-printed strontium-incorporated β-TCP bioceramic triply periodic minimal surface scaffolds with enhanced angiogenic and osteogenic properties
Source: Regen Biomater. 2025 Aug 12;12:rbaf080. doi: 10.1093/rb/rbaf080 (PMC12417083; doi:10.1093/rb/rbaf080)
Supplement: rbaf080_Supplementary_Data [file rbaf080_supplementary_data.zip › Supplementary_Information.docx]

**Supplementary Material:**

# Three-dimensional-printed strontium-incorporated β-TCP bioceramic triply periodic minimal surface scaffolds with enhanced angiogenic and osteogenic properties

Yanbo Shan^1,2,3†^, Yang Bai^2†^, Lisheng Zhao^2†^, Qing Zhou^4^, Shuo Yang^2^, Gang Wang^2^, Ye Lei^2^, Yuzheng Lu^3^, Yanbin Wu^3^, Yu Wei^3^, Jiang Peng^3^, Rujie He^4*^, Ning Wen^2*^, Bin Gu^2*^

^1^ Graduate School of the PLA General Hospital, Beijing 100853, China

^2^ Institute of Stomatology & Oral Maxilla Facial Key Laboratory, First Medical Center of Chinese PLA General Hospital, Beijing 100853, China

^3^ Institute of Orthopedics, Chinese PLA General Hospital, Beijing Key Lab of Regenerative Medicine in Orthopedics, Key Laboratory of Musculoskeletal Trauma & War Injuries PLA, Beijing 100853, China

^4^ Institute of Advanced Structure Technology, Beijing Institute of Technology, Beijing 100081, China

^†^Yanbo Shan, Yang Bai and Lisheng Zhao contributed equally to this work.

**^*^Corresponding Authors:**

Rujie He, Institute of Advanced Structure Technology, Beijing Institute of Technology, Beijing 100081, China; Email: herujie@bit.edu.cn.

Ning Wen, Institute of Stomatology & Oral Maxilla Facial Key Laboratory, First Medical Center of Chinese PLA General Hospital, Beijing 100853, China; Email: wenningchn@163.com.

Bin Gu, Institute of Stomatology & Oral Maxilla Facial Key Laboratory, First Medical Center of Chinese PLA General Hospital, Beijing 100853, China; Email: gubinmail301@163.com.


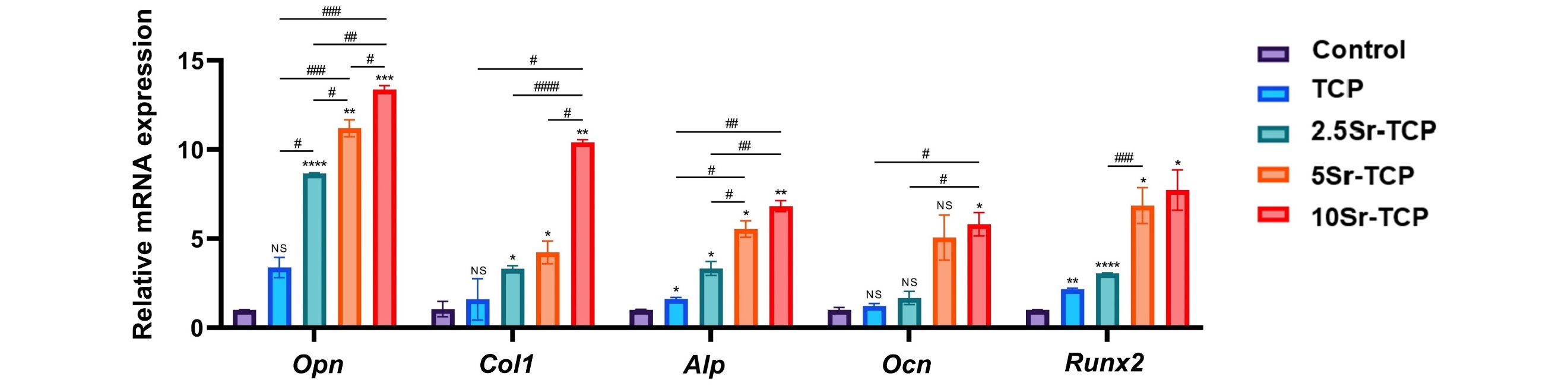
**Figure S1.** Relative mRNA expression of osteogenic genes (*Opn, Col1, Alp, Ocn, Runx2*) in MC3T3-E1 cells at 3 days. (n=3; ^NS^P>0.05, ^*^p<0.05, ^**^p<0.01, ^***^p<0.001, ^****^p<0.0001 versus control group; ^#^p<0.05, ^##^p<0.01, ^###^p<0.001, ^####^p<0.0001 among experimental groups).


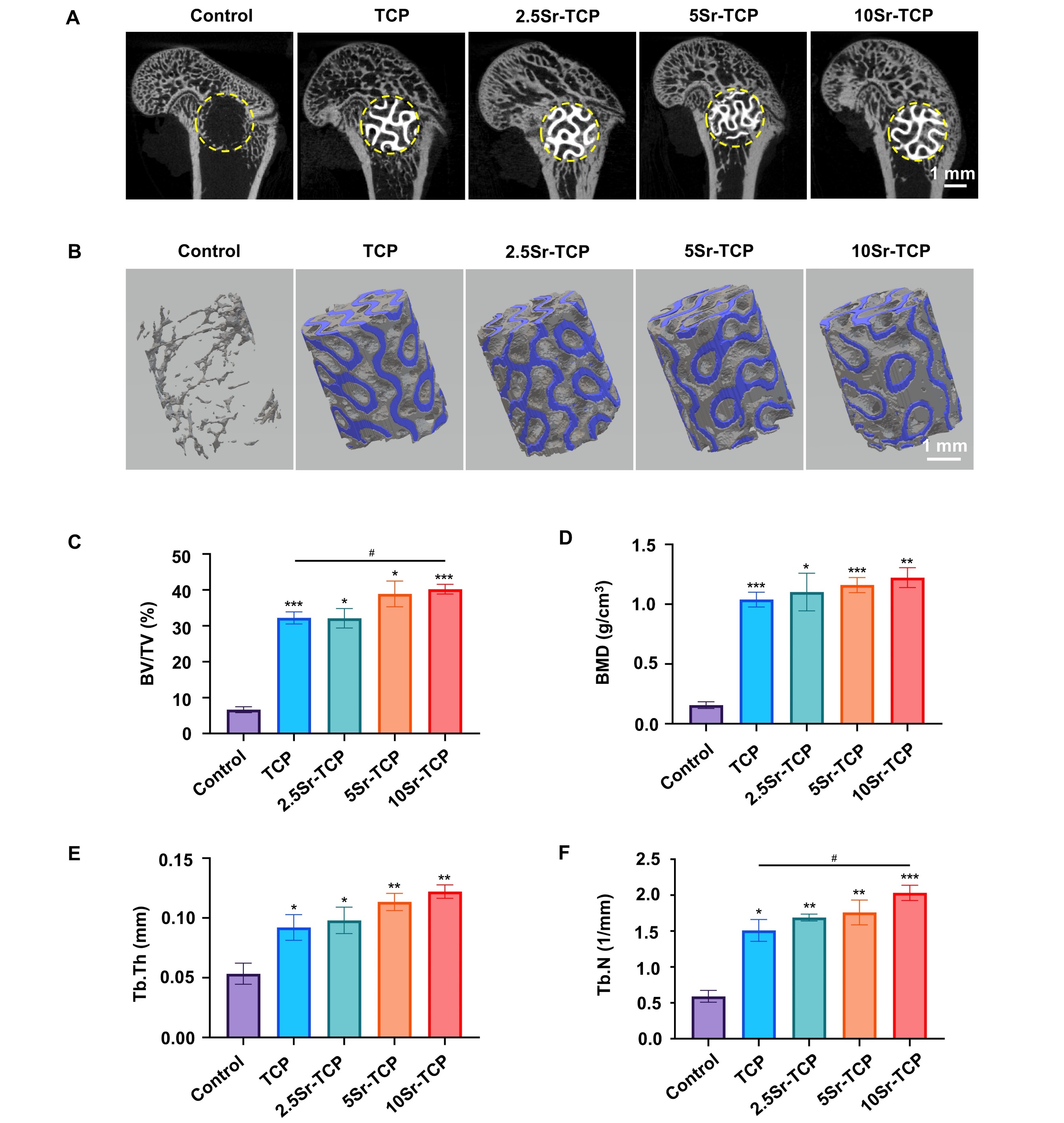
**Figure S2.** Micro-CT analysis of the impact of TCP and Sr-TCP scaffolds on in vivo bone regeneration. (**A**) Sagittal views and (**B**) 3D reconstructions of the rat femoral condylar defects treated with TCP, 2.5Sr-TCP, 5Sr-TCP, and 10Sr-TCP scaffolds after 4 weeks of implantation (Scaffolds: blue; Newborn bones: grey). (**C**) BV/TV, (**D**) BMD, (**E**) Tb.Th, and (**F**) Tb.N of the femoral condylar defects quantified through Micro-CT analysis (n=3; ^NS^P>0.05, ^*^p<0.05, ^**^p<0.01, ^***^p<0.001, ^****^p<0.0001 versus control group; ^#^p<0.05, ^##^p<0.01, ^###^p<0.001, ^####^p<0.0001 among experimental groups).

**
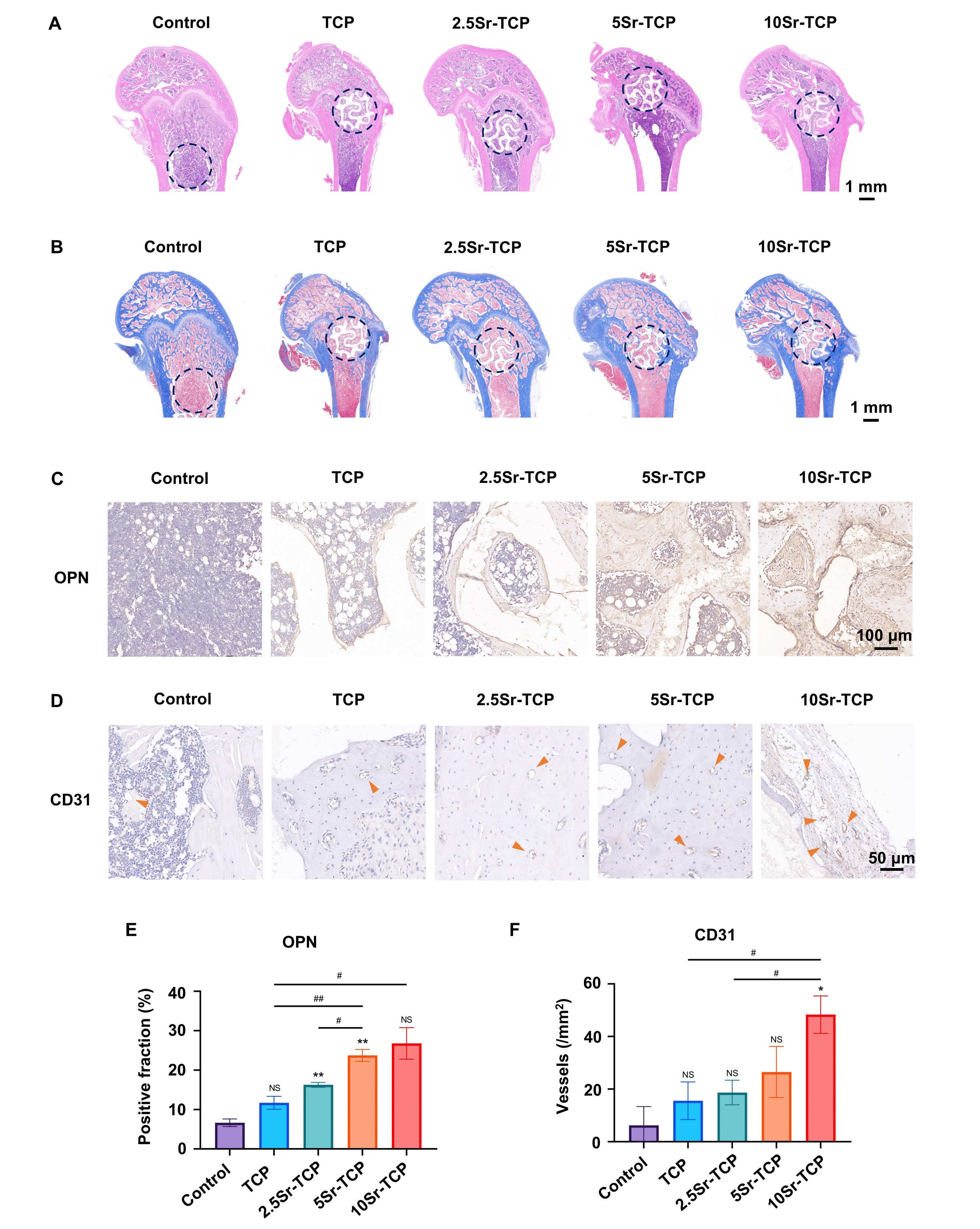
Figure S3.** Histological evaluation of bone regeneration in vivo. (**A**) H&E and (**B**) MT staining images of the defect areas in control, TCP, 2.5Sr-TCP, 5Sr-TCP, and 10Sr-TCP groups at 4 weeks post-implantation. (**C**) Immunohistochemical staining images of OPN, with (**E**) semi-quantitative analysis across groups. (**D**) Immunohistochemical staining images of CD31, with (**F**) semi-quantitative analysis of the vessels number across groups. Arrows point to the vessels. (n=3; ^NS^P>0.05, ^*^p<0.05, ^**^p<0.01 versus control group; ^#^p<0.05, ^##^p<0.01 among experimental groups).
